# Role of Parents in Body Mass Reduction in Children with Obesity—Adherence and Success of 1-Year Participation in an Intervention Program

**DOI:** 10.3390/medicina56040168

**Published:** 2020-04-09

**Authors:** Valentina Rahelić, Dominika Głąbska, Dominika Guzek, Eva Pavić, Ivana Rumora Samarin, Ana Bogdanić, Anita Špehar Uroić, Nataša Rojnić Putarek, Nevena Krnić

**Affiliations:** 1 Department of Nutrition and Dietetics, University Hospital Centre Zagreb, 12 Kišpatićeva Str., 10-000 Zagreb, Croatia; eva.pavic@kbc-zagreb.hr; 2 Chair of Dietetics, Department of Dietetics, Institute of Human Nutrition Sciences, Warsaw University of Life Sciences (WULS-SGGW), 159c Nowoursynowska Str., 02-787 Warsaw, Poland; dominika_glabska@sggw.pl; 3 Department of Food Market and Consumer Research, Institute of Human Nutrition Sciences, Warsaw University of Life Sciences (WULS-SGGW), 159c Nowoursynowska Str., 02-787 Warsaw, Poland; dominika_guzek@sggw.pl; 4 Faculty of Food Technology and Biotechnology, University of Zagreb, 6 Pierottijeva Str., 10-000 Zagreb, Croatia; irumora@pbf.hr; 5 Department of Pediatrics, University Hospital Centre Zagreb, 12 Kišpatićeva Str., 10-000 Zagreb, Croatia; ana.bogdanic@kbc-zagreb.hr; 6 Department of Pediatric Endocrinology and Diabetes, University Hospital Centre Zagreb, 12 Kišpatićeva Str., 10-000 Zagreb, Croatia; anita.spehar.uroic@kbc-zagreb.hr (A.Š.U.); nevena.krnic@kbc-zagreb.hr (N.K.); 7 Juraj Dobrila University of Pula, 30 Zagrebačka Str., 52-000 Pula, Croatia; nputarek@unipu.hr; 8 School of Medicine, University of Zagreb, 3 Šalata Str., 10-000 Zagreb, Croatia

**Keywords:** adolescents, preadolescents, obesity, body mass reduction program, dietary intervention, parents, meal consumption habits, multidisciplinary approach

## Abstract

*Background and Objectives*: Obesity in children and adolescents results in a number of serious health-related consequences necessitating early treatment. Support from family members and family-focused lifestyle interventions can improve effectiveness of the treatment. The aim of the study was to assess the effects of parental characteristics and family-based dietary habits on the adherence and success of a body mass reduction program in children with obesity included in a lifestyle intervention program after 1 year. *Materials and Methods*: The program included dietetic, psychosocial, and endocrine counseling given to individuals either alone or in groups and was conducted by a multidisciplinary team (consisting of endocrinologists, nurses, psychologists, social counselors, dietitians, and physiotherapists). A total of 113 children aged 10–17 years (mean age 12.9 ± 2.0; 60 girls, 53 boys) were included in the program. After 1 year of participation, the rate of adherence and success were assessed. The effect of the participants’ general characteristics, including anthropometric data, as well as parental characteristics (marital status, employment, education, body mass index (BMI), duration of breastfeeding) and the circumstances of meal consumption (eating at home or outside, fast food consumption), was analyzed. *Results*: The most important factors predicting body mass reduction success were baseline BMI (*p* < 0.0001) and waist–hip ratio (WHR) (*p* = 0.04), but they did not predict body mass reduction adherence. *Conclusions*: The meal consumption habits and support from family members may be among the determinants of adherence to a body mass reduction program for preadolescents and adolescents with obesity. However, the results of the presented study suggested that baseline BMI and WHR are the most important determinants of the body mass reduction success.

## 1. Introduction

According to the World Health Organization (WHO) [1], proper nutrition is essential for survival, physical growth, mental development, performance, productivity, health, and well-being across the life span of individuals. Childhood nutrition especially has a lifelong effect, so lack of a properly balanced diet, including excessive energy intake, in children and adolescents is a serious concern in developed countries. The Global Strategy on Diet, Physical Activity and Health developed by the WHO addressed the increasing prevalence and burden of non-communicable diet-related diseases [2]. The global public health policy of the WHO that was represented in the Global Targets 2025 included the goal to reduce the prevalence of childhood overweight before the year 2025 [3]. In addition, the Commission on Ending Childhood Obesity of the WHO emphasized the necessity to prevent development of obesity, as well as to treat the existing obesity in children and adolescents [4].

As estimated by the WHO, 170 million children are overweight or obese [5], and the frequency of excessive body mass is increasing every year [6], resulting in a number of serious health-related consequences, including cardiovascular diseases [7], type 2 diabetes [8], and increased risk of cancer during adulthood [9]. Moreover, children with obesity experience numerous psychosocial consequences that are associated with obesity stigma, including teasing and bullying by their peers, teachers, and society [10]. It is also identified that children with obesity have a poor overall quality of life compared to their lean peers [11].

The interventions aiming at body mass reduction may be helpful to reduce the negative consequences of excessive body mass as long as the lifestyle modification is continued [12]. A number of studies analyzing body mass reduction programs indicated that programs including planned dietary intervention [13] and physical activity [14], including in-hospital multidisciplinary integrated programs, are effective [15], but for motivation towards body mass reduction, relatives are especially important [16]. When family members participate in the program, it is observed that even if child’s adiposity is not reduced, obesogenic health behaviors and parents’ feeding habits improve [17], so increasing awareness of the importance of following a properly balanced diet and regular physical activity may be crucial [18]. Similarly, physical activity programs conducted in groups of peers may be effective [19], while for pediatric patients, positive predictors of successful body mass reduction include male gender, older age, and smaller waist circumference [20].

The Cochrane review by Mead et al. [21] indicated that multi-component behavior-changing interventions that incorporate diet, physical activity, and behavior change may be beneficial in achieving only small, short-term reductions in body mass index (BMI) and weight in children aged 6 to 11 years. At the same time, an updated Cochrane review by Al-Khudairy et al. [22] emphasized that for treatment of overweight or obese adolescents aged 12 to 17 years, evidence that multidisciplinary interventions involving a combination of diet, physical activity, and behavioral components reduce the BMI is of a low quality, with inconsistent results, and feature a risk of bias.

However, both family and household environment may influence the body mass of children [23], as it was confirmed in a recent systematic review [24], and thus they may have an impact on the effectiveness of the intervention [25]. Therefore, the aim of the present study was to assess the effects of parental characteristics and family-based dietary habits on the adherence and success of a body mass reduction program in children with obesity after 1 year of intervention.

## 2. Materials and Methods

### 2.1. Ethical Statement

This study was conducted at the Department of Nutrition and Dietetics and the Department of Pediatric Endocrinology and Diabetes, University Hospital Center Zagreb, Zagreb, Croatia. The program involved individual and group dietetic, psychosocial, and endocrine counseling given to children and their parents/legal guardians. It was conducted by a multidisciplinary team (consisting of endocrinologists, nurses, psychologists, social counselors, dietitians, and physiotherapists).

The study followed the guidelines of the Declaration of Helsinki, and the procedures were approved by the Ethics Committee of the University Hospital Center Zagreb (No. 8.1–19/183–3–02/21AG; 19.07.2019). All the participants and their parents/legal guardians provided their written informed consent to participate in the body mass reduction program and in the study assessing its effectiveness.

### 2.2. Study Group

The studied group consisted of patients recruited from the Department of Pediatric Endocrinology and Diabetes, University Hospital Center Zagreb, who participated in the body mass reduction program conducted from 2013 to 2018. The children meeting the following inclusion criteria who visited the clinic at least once in six months were included in the study:

- Participants of the body mass reduction program;
- Croatian-speaking children and parents/legal guardians;
- Preadolescents and adolescents (over 10 years old [26,27,28]);
- Obesity diagnosed based on the BMI value ≥ 95th percentile as recommended by the Centers for Disease Control and Prevention (CDC) [29] for the gender-and age-dependent growth reference cutoffs for children and adolescents by the CDC [30] and related software [31,32];
- Written informed consent to participate in the study.

The exclusion criteria were as follows:

- Age over 17 years (attributed to being adult after 1 year of the dietary intervention);
- Any chronic diet-related diseases other than those diagnosed as resulting from the excessive body mass (hypertension, dyslipidemias, disturbed glucose metabolism, and polycystic ovary syndrome);
- Any genetic syndromes and endocrine disorders resulting in obesity;
- Lack of regular dietitian visits with body mass control at least once in 6 months;
- Any missing baseline data associated with refused information about parental characteristics (marital status, employment, education, BMI, duration of breastfeeding) and the circumstances of meal consumption (eating at home or outside, fast food consumption).

The number of 113 children aged 10–17 years was included into the study (mean 12.9 ± 2.0; 53.1% female). The number of children participating in the body mass reduction program was limited mainly by the will of children and their parents or legal guardians to participate; in spite of the fact that the program was free of charge for the participants, some patients of the Department of Pediatric Endocrinology and Diabetes were not willing to participate. Moreover, a number of them did not meet either the inclusion criteria or the criterion of regular visits in the clinic at least once in six months. Finally, out of 146 children participating in the body mass reduction program, the number of 113 children was included to the study based on inclusion/exclusion criteria.

The characteristics of the studied participants were as follows: 49 preadolescents aged 10–12 years (mean age 11.1 ± 0.8, 53.1% female) and 64 adolescents aged 13–17 years (mean age 14.3 ± 1.3, 53.1% female). Their baseline BMI percentile was characterized by the median of 99 (varied from 95 to 100—nonparametric distribution, verified using the Shapiro–Wilk test).

### 2.3. Body Mass Reduction Program

The body mass reduction program was organized and conducted in the following: the Department of Pediatric Endocrinology and Diabetes, University Hospital Center Zagreb; the Croatian Referral Center for Pediatric Endocrinology and Diabetes of the Ministry of Health, Republic of Croatia; and the Department of Nutrition and Dietetics, University Hospital Center Zagreb. A multidisciplinary approach was followed in the pediatric outpatient clinic for the treatment of pediatric patients with obesity by a team consisting of endocrinologists, nurses, psychologists, and social counselors from the Department of Pediatric Endocrinology and Diabetes in cooperation with dietitians from the Department of Nutrition and Dietetics and physiotherapists from the Clinic for Rheumatic Diseases and Rehabilitation, University Hospital Center Zagreb.

Within the program, the participants were recruited by their endocrinologists. The program commenced with an intensive 1-week training for children and their parents/legal guardians which was conducted in groups consisting of not more than 10 children. The groups were designed to be age-homogenous to achieve better social connections within the peer groups. During the program, the participants attended the outpatient clinic to, inter alia, consume individually planned and properly balanced meals.

During the 1-week group training, both children and their parents/legal guardians participated in predetermined daily courses consisting of 26 h of lessons in total conducted by endocrinologists (4 h), dietitians (8 h), psychologists (5 h), social counselors (4 h), and physiotherapists (5 h, including organized physical activity for children). Some of the lectures were delivered to both children and their parents/legal representatives, while others were delivered to separate groups. Besides group counseling, during the 1-week course, the participants were also provided with individual counseling, if needed.

After 1 week of group training, children and their parents/legal guardians had the control meetings with appropriate anthropometric measurements obtained through 1-day visits once a month for the first 6 months and then every 2 months for the next 6 months (with biochemical measurements taken every 6 months).

During each consultation, a dietitian took anthropometric measurements and analyzed the current dietary problems, as each child had received after group training an individually planned dietary program and diet recommendations based on the changes in dietary habits. The recommendations were not based on the calorie count, but on the lower fat intake and a smaller portion size, as generally recommended [33], and the approach was based on the Mediterranean diet model, as recommended by the Croatian standard guidelines for hospital nutrition [34]. The following advice was given in addition to dietary recommendations: need of breakfast consumption and planned family meal consumption; reduction of intake of sugar-sweetened beverages and highly processed products rich in calories and fat; reduction of the frequency of eating out, especially of the fast food; and increase in the intake of fruits and vegetables. It was insisted that proper serving sizes must be followed, especially for crucial foods, such as milk and dairy products, fruits, and vegetables, which are sources of calcium and fiber, as well as proper proportions of proteins, fats, and carbohydrates were advised, while relevant examples of meals were presented.

The BMI percentile in the studied group after 12 months of the body mass reduction program was characterized by the median of 98 (varied from 79 to 100—nonparametric distribution, verified using the Shapiro–Wilk test).

### 2.4. Assessed Variables

**Figure 1:**
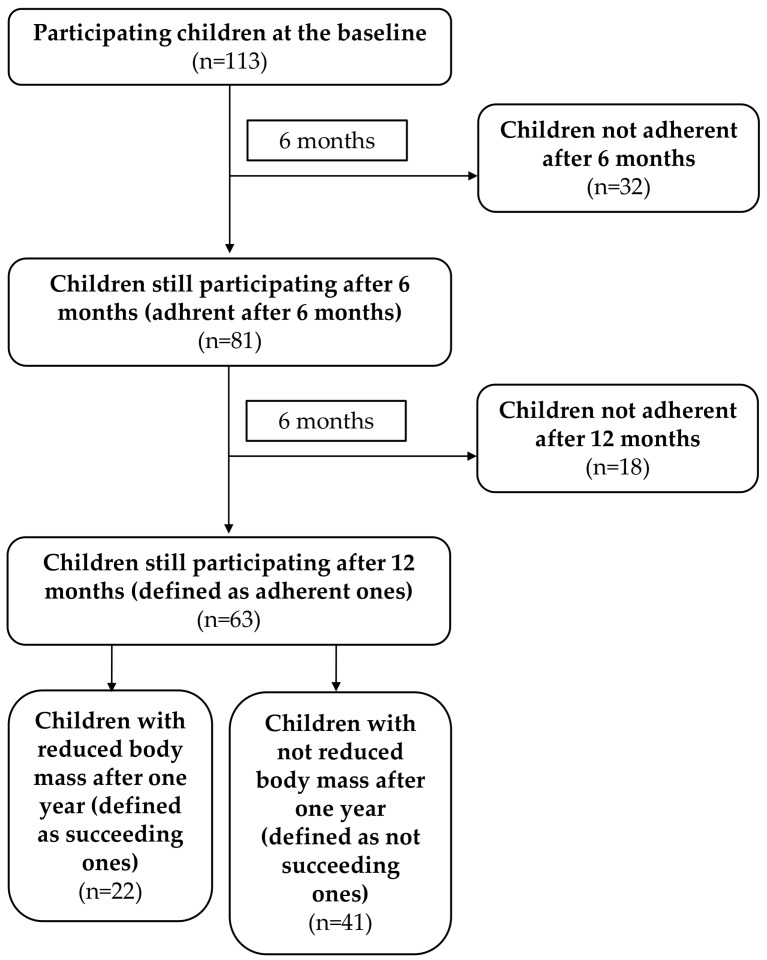
The flowchart of the study group; n—number of children.

After one year of participation in the body mass reduction program, the adherence and success of the program were assessed. Adherence was defined as participation in the program for a year attending clinic/dietetic counseling at least once in 6 months and it was verified for all the included participants after 1 year of the program participation (113 participants divided into subgroups of adherent ones—63 participants and non-adherent ones—50 participants). Successful weight loss was determined based on the decrease in the BMI percentile and it was verified for the subgroups of participants adherent after 1 year of the program participation (63 participants divided into subgroups of successful ones—22 participants and not successful ones—41 participants). Figure 1 presents the flowchart of the study with groups and related subgroups.

The measurements of body mass and height were taken by a dietitian according to a widely applied methodology [35] using a calibrated weighing scale with an accuracy of ± 0.1 kg and a stadiometer with an accuracy of ± 0.5 cm. The BMI was calculated using the Quetelet’s equation (body mass (kg)/height^2^ (m^2^)), and the BMI percentile was assessed using the gender- and age-dependent growth reference cutoffs for children and adolescents suggested by the CDC [20] and related software [31,32], which were applied to indicate the specific percentile. A decreased BMI percentile was defined as a difference of more than one percentile between percentiles at the beginning of the program and after 1 year.

The additional measurements obtained in the study group included the waist–hip ratio (WHR). Both waist and hip circumferences were measured by a dietitian using a non-elastic flexible measuring tape with an accuracy of ± 0.5 cm. After that, the WHR was calculated by dividing the value of the waist circumference by the value of the hip circumference [36].

At inclusion into the program, the participants were required to fill a questionnaire with open-ended and closed-ended questions evaluating the following data:

- Family’s place of residence (an open-ended question with the answer attributed to one of the following categories: village, city);
- Parental marital status (a closed-ended question with the following possible answers: married, in a marriage-like relationship, separated, widowed, single; these were clustered afterwards into married/in a marriage-like relationship, separated/widowed/single);
- Father’s and mother’s employment status (a closed-ended question with the following possible answers: unemployed, employed);
- Father’s and mother’s education (a closed-ended question with the following possible answers: primary, secondary, vocational, higher, postgraduate; these were clustered afterwards into secondary or lower, higher);
- Father’s and mother’s body mass and height (an open-ended question; the answers were used to calculate the BMI using the Quetelet’s equation);
- Duration of breastfeeding in months (an open-ended question);
- Number of meals consumed at home (an open-ended question about the number of meals per week);
- Number of meals consumed outside (an open-ended question about the number of meals per week);
- Number of fast-food meals (an open-ended question about the number of meals (with adequate examples of burgers, pizza, hot dogs, etc.) per week);
- Number of snacks consumed (an open-ended question about the number of snacks defined as salty processed snacks other than nuts with adequate examples of chips, crisps, crackers, etc. per week);
- Breakfast consumption (a closed-ended question with the following answers: typically no, typically yes);
- Place of breakfast consumption (an open-ended question with the answer attributed to the following categories: home, school, others; multiple answers were allowed);
- Lunch consumption (a closed-ended question with the following answers: typically no, typically yes);
- Place of lunch consumption (an open-ended question with the answer attributed to one of the following categories: home, school, others; multiple answers were allowed; the place of lunch preparation was not taken into account);
- Dinner consumption (a closed-ended question with the following answers: typically no, typically yes);
- Place of dinner consumption (an open-ended question with the answer attributed to one of the following categories: home, grandmother’s house; multiple answers were allowed).

### 2.5. Statistical Analysis

The distributions were verified using the Shapiro–Wilk test. The subgroups were compared using the χ^2^ test, the Student’s *t*-test (for parametric distributions), and the Mann–Whitney *U*-test (for nonparametric distributions).

The statistical analysis was conducted using Statistica v.13.3 (StatSoft Inc., Tulsa, OK, USA) with a logistic regression module and Statgraphics Plus for Windows v.5.1 (Statgraphics Technologies Inc., The Plains, VA, USA). The *p* ≤ 0.05 was accepted as statistically significant.

## 3. Results

### 3.1. Characteristics of the Studied Group 

The baseline characteristics of the studied group of preadolescents and adolescents combined recorded for the assessment of program adherence are presented in Table 1. The general characteristics (gender, age, place of residence, WHR, and BMI) were not found to differ between the participants who were or were not adherent to the intervention program for 1 year.

The baseline characteristics of the studied group of preadolescents and adolescents combined recorded for the assessment of success of the body mass reduction program are presented in Table 2. The general characteristics (gender, age, and place of residence) were not found to differ between the participants who were or were not successful in body mass reduction verified after 1 year of participation in the intervention program. Individuals from the successful weight loss group were characterized by lower WHR (*p* = 0.04) and BMI values at the baseline (*p* < 0.0001) than those who did not succeed.

### 3.2. Determinants of Program Adherence

The parents-related determinants of program adherence of preadolescents and adolescents combined are presented in Table 3. None of the assessed variables were found to influence program adherence. However, a smaller share of separated/widowed/single parents in the group of adherent children was observed compared to the non-adherent group, suggesting that children with a single caregiver had lower adherence, although this difference was not statistically significant (*p* = 0.09).

The determinants of program adherence related to meal consumption habits of preadolescents and adolescents combined are presented in Table 4. None of the assessed variables were found to influence program adherence except for the place of breakfast consumption. The highest share of participants who were adherent after 1 year of intervention declared that they had their breakfast in places other than home and school (*p* = 0.01). A similar situation was observed for breakfast consumption at home, although this difference was not statistically significant (*p* = 0.08).

### 3.3. Determinants of Success of Body Mass Reduction

The parents-related determinants of success of body mass reduction of preadolescents and adolescents combined are presented in Table 5. No differences were observed in the assessed variables between the successful and the unsuccessful groups. However, a longer duration of breastfeeding of children in the group of participants who were successful in body mass reduction compared to the unsuccessful group was stated, although this difference was not statistically significant (*p* = 0.09).

The determinants of success of body mass reduction that were related to meal consumption habits of preadolescents and adolescents combined are presented in Table 6. No differences were observed in the assessed variables between the successful and the unsuccessful groups. However, increased consumption of fast food among the participants with unsatisfactory body mass reduction (up to 20 servings per week) compared to the participants who demonstrated successful body mass reduction (less than three servings) was stated, although this difference was not statistically significant (*p* = 0.09).

## 4. Discussion

### 4.1. Determinants of Program Adherence

Adherence, being crucial to obtain a long-lasting effect of body mass reduction, is found to generally be lower if the duration of the weight management program is longer [37]. Moreover, it has been suggested that to achieve a positive outcome, any intervention program should last at least 6 months [38]. In the present study, which presents 12 months of such a program, it was found that only minor influencing factors were observed for program adherence.

It was stated that the participants who were adherent to the program more often ate their breakfast outside their homes or schools. No other variables evaluated were found to be related to adherence in the presented study. However, the potential role of the mother’s BMI must be emphasized here. The systematic review and meta-analysis by Heslehurst et al. [39] indicated a higher prevalence of childhood obesity and the meta-analysis by Castillo-Laura et al. [40]—a higher prevalence of high adiposity in the children whose mothers are obese. In the meta-analysis of individual data by Voerman et al. [41], an association was shown between childhood obesity and the mother’s weight gain during gestation. Not only are the biological influences of weight loss or weight gain during pregnancy important, as these affect the birth weight [42], but the general nutritional behaviors of mothers are important as well, as these affect the nutritional behaviors of their children [43].

The association between the nutritional behaviors of mothers and their children is related to a number of determinants of the children’s eating behaviors, while the most important ones are those conditioned by parents and family [44]. Thus, nutritional preferences of mothers generally influence preferences [45] and intake [46] of their children. Such associations may explain the consequent association between the body mass status of mothers and their offspring. In the Fels Longitudinal Study cohort, a western cohort from the United States of America, this relationship was stated to be stronger for daughters than for sons, as well as to be observed earlier in the life of daughters than sons [47]. However, in an Asian cohort of Chinese respondents, this association was observed only after birth and it was mild [48]. Therefore, it may be concluded that such associations may be dependent on the country, or even on the ethnicity.

The influence of parents on eating behaviors of their children may explain the association between the place of breakfast consumption and the children’s adherence to the body mass reduction program observed in the presented study. Based on the results found, it may be supposed that at-home breakfast consumption (or consumption at the grandmother’s home, or while going to school, but not at school) is typical for children from well-motivated families. Taking this into account, the role of family in assisting in the body mass reduction of children must be emphasized from enrollment through every aspect of a weight management program.

As observed in a population-based longitudinal study by Parkinson et al. [49], mothers do not tend to perceive their children as overweight or obese, and they accept their children as having a problem with excessive body mass only in extreme cases. As a result, a number of parents of children with obesity do not want their child to lose weight [50], which may reduce effectiveness of a body mass reduction program, as the children whose parents do not perceive them as overweight or obese are less likely to view their body mass negatively or to actively try to lose weight [51]. Especially in the case of adolescents with obesity, it was observed that the effect of a body mass reduction program was associated with their beliefs regarding a number of issues, including their body mass control, potential difficulties in body mass reduction, potential medical and family-related reasons for excessive body mass, and particularly their beliefs regarding willingness of their family members to participate in their diet plan [52]. Consequently, to attain the goal of weight loss in children, parents must be engaged in the whole process and participate; otherwise, body mass reduction may be very hard to achieve, or even become impossible.

### 4.2. Determinants of Success of Body Mass Reduction

The success rate of weight management programs is generally reported to be low [53]. Unhealthy eating habits are often reported among female children and parents with a lower level of education [54,55,56], while in some studies, better rate of success was shown to be related to a higher level of mother’s education [57,58]. However, in the presented study, such a relationship was not observed.

It was found that the baseline WHR and BMI differed between the children who were successful and unsuccessful in body mass reduction. The WHR and BMI were lower in the group of successful children, suggesting that the type of body composition and the body mass itself might influence weight reduction, primarily in the case of abdominal obesity [59]. Such minor influence of the assessed factors is in agreement with the results of Donkor et al. [60]. At the same time, in the analyzed group, not only there was only one major determinant (baseline body mass), but it determined only success and not adherence.

It must be emphasized that the study was conducted in a relatively homogenous group characterized by the BMI percentile of 95 or higher. Taking it into account, it must be indicated that even in such a group the baseline BMI matters and it may determine success of body mass reduction. It may allow indicating a group especially prone for successful body mass reduction as adolescents with obesity and a little bit lower BMI.

Based on the obtained results indicating that the conducted family-based intervention may be effective in some participants, it must be emphasized that parents and family play a role in the body mass reduction of children with obesity, family-based interventions may be an appropriate tool for achieving body mass reduction in children [61]. The program used in the presented study was such an intervention, as both children with obesity and their parents participated in group sessions and individual training. According to the literature, such an approach is more cost-effective compared to other models [62], especially if families in which both parents and children are obese are targeted [63]. Sustained monitoring and goal setting, healthy peer interactions, and support from family as well as the home environment are indicated as the main determinants of effectiveness of such approaches [64], which once again emphasize the role of family members.

However, not only children may lose weight while participating in such family-based interventions; body mass reduction may also be observed in parents [65], which in turn may predict favorable outcomes in children [66]. Some authors even indicate that parental weight changes are the key predictors of the weight changes in children [67]. Based on the results presented in the abovementioned studies, it can be emphasized that evaluating parental body mass in body mass reduction programs is important, as the excessive body mass of parents (maybe not recognized by themselves) may be related to the lack of recognition of their child’s excessive body mass and lack of adherence and support to such programs.

However, such results were not observed in the presented study. The most important factors predicting body mass reduction success here were the baseline BMI and WHR, but they did not predict body mass reduction adherence. Those observations may be important for dieticians and practitioners, as they should be aware that a baseline problem may determine results of body mass reduction, as well as that adherence is not always associated with success in the end.

In spite of the fact that the results of the presented study may be important for planning further body mass reduction interventions, some limitations of the study must also be mentioned. The main limitation is associated with the sample size and variability of the included participants being both boys and girls, both preadolescents and adolescents. As a result, the risk of false discoveries based on the number of performed analyses must be mentioned and the observations need to be verified in larger samples of participants. Moreover, the studied participants were observed only during one year of the intervention and it was not verified how their body mass changes after the participation in the program. At the same time, as a measure of successful body mass reduction, a decreased BMI percentile was chosen, and was defined as a difference of more than one percentile between percentiles at the beginning of the program and after 1 year, but it is not the only possible approach, as some authors rather use the BMI standard deviation score [68], and other suggest that not the BMI, but the body composition should rather be used [69], so it may be the other limitation. The other issue is the fact that self-reported variables are prone to the risk of bias, which is especially important in the case of such variables as reported parental weight and height [70]. Last but not least, there may have also been other predictors of body mass reduction adherence and success (such as a diet followed during the intervention), including family-related predictors, while only some of them were included to the analysis.

## 5. Conclusions

The meal consumption habits and support from family members may be among the determinants of adherence to a body mass reduction program for preadolescents and adolescents with obesity. However, the results of the presented study suggested that the baseline BMI and WHR are more important determinants of body mass reduction success.

## Figures and Tables

**Table 1:** The baseline characteristics of the studied group considered for the assessment of program adherence (n = 113).

Characteristics		No Adherence Verified after 1 Year of Intervention (n = 50)	Adherence Verified after 1 Year of Intervention (n = 63)	*p*
Gender n (%)	Male	25 (50.0)	35 (55.6)	0.69
	Female	25 (50.0)	28 (44.4)	
Age (years)	Mean ± SD	13.1 ± 2.0	12.6 ± 1.9	0.25
	Median (range)	13 * (10–17)	13 * (10–16)	
Place of residence n (%)	Village	10 (20.0)	19 (30.2)	0.31
	City	40 (80.0)	44 (69.8)	
WHR ^1^	Mean ± SD	0.97 ± 0.06	0.95 ± 0.06	0.41
	Median (range)	0.97 (0.85–1.1)	0.96 (0.78–1.1)	
BMI percentile	Mean ± SD	98.35 ± 1.08	98.43 ± 1.01	0.74
	Median (range)	99 * (95–100)	99 * (95–100)	
BMI *z*-score	Mean ± SD	2.14 ± 0.30	2.12 ± 0.26	0.70
	Median (range)	2.17 (1.54–2.85)	2.10 (1.44–2.71)	

* nonparametric distribution (verified using the Shapiro–Wilk test; *p* ≤ 0.05); ^1^ any missing data for respondents in the subgroup; SD—standard deviation; n—number of children; WHR—waist–hip ratio; BMI—body mass index.

**Table 2:** Baseline characteristics of the studied group recorded for the assessment of the success of the body mass reduction program (verified after 1 year of participation in the intervention program) (n = 63).

Characteristics		No Successful Weight Loss (n = 41)	Successful Weight Loss (n = 22)	*p*
Gender n (%)	Male	20 (48.8)	15 (68.2)	0.23
	Female	21 (51.2)	7 (31.8)	
Age (years)	Mean ± SD	13.1 ± 1.8	13.1 ± 2.4	0.70
	Median (range)	13 * (10–17)	13 * (10–17)	
Place of residence n (%)	Village	11 (26.8)	8 (36.4)	0.62
	City	30 (73.2)	14 (63.6)	
WHR ^1^	Mean ± SD	0.97 ± 0.06	0.93 ± 0.07	0.04
	Median (range)	0.97 (0.81–1.06)	0.94 (0.78–1.06)	
BMI percentile	Mean ± SD	98.80 ± 0.75	97.73 ± 1.08	< 0.0001
	Median (range)	99 * (96–100)	98 * (95–99)	
BMI *z*-score	Mean ± SD	2.23 ± 0.23	1.93 ± 0.18	< 0.0001
	Median (range)	2.21 (1.71–2.71)	1.97 (1.44–2.24)	

* nonparametric distribution (verified using the Shapiro–Wilk test; *p* ≤ 0.05); ^1^ any missing data for respondents in the subgroup; SD—standard deviation; n—number of children; WHR—waist–hip ratio; BMI—body mass index.

**Table 3:** The parents-related determinants of program adherence for all the program participants.

Characteristics		No Adherence Verified after 1 Year of Intervention (n = 50)	Adherence Verified after 1 Year of Intervention (n = 63)	*p*
Parents’ marital status ^1,2^	married/in a marriage-like relationship	33 (68.8)	52 (83.9)	0.09
	separated/widowed/single	15 (31.3)	10 (16.1)	
Father’s employment ^1,2^	Unemployed	4 (8.9)	5 (8.2)	0.82
	Employed	41 (91.1)	56 (91.8)	
Mother’s employment ^1,2^	Unemployed	7 (14.9)	17 (27.4)	0.18
	Employed	40 (85.1)	45 (72.6)	
Father’s education ^1,2^	Secondary or lower	33 (76.7)	46 (75.4)	0.94
	Higher	10 (23.3)	15 (24.6)	
Mother’s education ^1,2^	Secondary or lower	30 (65.2)	42 (68.8)	0.85
	Higher	16 (34.8)	19 (31.2)	
Father’s BMI ^1^ (kg/m^2^)	Mean ± SD	31.6 ± 5.6	30.3 ± 5.2	0.27
	Median (range)	30.6 * (21.2–47.3)	29.9 (19.1–41.0)	
Mother’s BMI ^1^ (kg/m^2^)	Mean ± SD	27.5 ± 5.6	27.3	0.79
	Median (range)	26.3 * (19.8–44.1)	25.8 * (18.4–41.0)	
Breastfeeding (months) ^1^	Mean ± SD	7.1 ± 7.6	8.0 ± 9.7	0.79
	Median (range)	5 * (0–36)	5.5 * (0–38)	

* nonparametric distribution (verified using the Shapiro–Wilk test; *p* ≤ 0.05); ^1^ any missing data for respondents in the subgroup; SD—standard deviation; n—number of children; BMI—body mass index; ^2^ expressed as number (percentage).

**Table 4:** Meal consumption habits for all the program participants—determinants of program adherence.

Characteristics		No Adherence Verified after 1 Year of Intervention (n = 50)	Adherence Verified after 1 Year of Intervention (n = 63)	*p*
Meals at home ^1^ (meals/week)	Mean ± SD	20.2 ± 8.1	20.2 ± 7.1	0.89
	Median (range)	21 * (0–35)	21 * (0–35)	
Meals outside ^1^ (meals/week)	Mean ± SD	6.2 ± 4.2	5.8 ± 4.1	0.47
	Median (range)	5 * (0–20)	5 * (0–21)	
Fast food outside ^1^ (meals/week)	Mean ± SD	1 ± 1.3	0.8 ± 2.6	0.12
	Median (range)	0.5 * (0–5)	0 * (0–20)	
Snacks ^1^ (servings/week)	Mean ± SD	1.7 ± 2.4	1.0 ± 1.2	0.16
	Median (range)	1 * (0–15)	1 * (1–7)	
Breakfast consumption ^1^	No	3 (6.1)	5 (8.2)	0.94
	Yes	47 (93.9)	56 (91.8)	
Place of breakfast consumption ^1,2^	At home	34 (53.1)	42 (56.8)	0.08
	At school	30 (46.9)	23 (31.1)	0.86
	Others	0	9 (12.2)	0.01
Lunch consumption ^1^	No	0	1 (1.6)	0.92
	Yes	50 (100.0)	60 (98.4)	
Place of lunch consumption ^1,2^	At home	44 (75.9)	56 (81.2)	0.61
	At school	14 (24.1)	11 (15.9)	0.35
	Others	0 (0)	2 (2.9)	0.55
Dinner consumption ^1^	No	0	2 (3.3)	0.56
	Yes	50 (100.0)	59 (96.7)	
Place of dinner consumption ^1,2^	At home	46 (86.8)	59 (95.2)	0.21
	At grandmother’s house	7 (13.2)	3 (4.8)	

* nonparametric distribution (verified using the Shapiro–Wilk test; *p* ≤ 0.05); ^1^ any missing data for respondents in the subgroup; ^2^ multiple-choice question; SD—standard deviation; n—number of children.

**Table 5:** The parents-related determinants of success of body mass reduction for all the program participants.

Characteristics		No Successful Weight Loss (n = 41)	Successful Weight Loss (n = 22)	*p*
Parents’ marital status ^1,2^	married/in a marriage-like relationship	35 (87.5)	17 (77.3)	0.49
	separated/widowed/single	5 (12.5)	5 (22.7)	
Father’s employment ^1,2^	Unemployed	4 (10.3)	1 (4.5)	0.77
	Employed	35 (89.7)	21 (95.5)	
Mother’s employment ^1,2^	Unemployed	12 (30.0)	5 (22.7)	0.75
	Employed	28 (70.0)	17 (77.3)	
Father’s education ^1,2^	Secondary or lower	29 (74.4)	17 (77.3)	0.96
	Higher	10 (25.6)	5 (22.7)	
Mother’s education ^1,2^	Secondary or lower	28 (71.8)	14 (63.6)	0.71
	Higher	11 (28.2)	8 (36.4)	
Father’s BMI ^1^ (kg/m^2^)	Mean ± SD	30.2 ± 5.3	30.3 ± 5.0	0.96
	Median (range)	29.9 (19.1–39.2)	28.7 (23.4–42.0)	
Mother’s BMI ^1^ (kg/m^2^)	Mean ± SD	28.1 ± 6.3	25.9 ± 5.1	0.13
	Median (range)	27.5 (18.4–41.0)	24.4 * (20.6–41.0)	
Breastfeeding (months) ^1^	Mean ± SD	5.7 ± 5.9	10.0 ± 9.6	0.09
	Median (range)	3 * (0–24)	8 * (0–36)	

* nonparametric distribution (verified using the Shapiro–Wilk test; *p* ≤ 0.05); ^1^ any missing data for respondents in the subgroup; ^2^ expressed as number (percentage); SD—standard deviation; n—number of children; BMI—body mass index.

**Table 6:** Meal consumption habits for all the program participants —determinants of success of body mass reduction.

Characteristics		No Successful Weight Loss (n = 41)	Successful Weight Loss (n = 22)	*p*
Meals at home ^1^ (meals/week)	Mean ± SD	20.8 ± 7.8	19.0 ± 5.5	0.46
	Median (range)	21 * (0–35)	16.8 (9–30)	
Meals outside ^1^ (meals/week)	Mean ± SD	5.5 ± 4.0	6.3 ± 4.5	0.49
	Median (range)	5 * (0–15)	5 * (0–21)	
Fast food outside ^1^ (meals/week)	Mean ± SD	0.9 ± 3.1	0.7 ± 0.8	0.09
	Median (range)	0 * (0–20)	1 * (0–3)	
Snacks ^1^ (servings/week)	Mean ± SD	0.9 ± 0.9	1.4 ± 1.6	0.13
	Median (range)	1 * (0–3)	1 * (0–7)	
Breakfast consumption ^1^	No	4 (9.8)	1 (5.0)	0.89
	Yes	37 (90.2)	19 (95.0)	
Place of breakfast consumption ^1,2^	At home	29 (60.4)	13 (50)	0.54
	At school	13 (27.1)	10 (38.5)	0.45
	Others	6 (12.5)	3 (11.5)	0.80
Lunch consumption ^1^	No	40 (97.6)	20 (100.0)	0.71
	Yes	1 (2.4)	0	
Place of lunch consumption ^1,2^	At home	39 (84.8)	17 (77.3)	0.67
	At school	6 (13.0)	4 (18.2)	0.84
	Both	1 (2.2)	1 (4.5)	0.82
Dinner consumption ^1^	No	0	2 (10.0)	0.19
	Yes	41 (100.0)	18 (90.0)	
Place of dinner consumption ^1,2^	At home	41 (93.2)	18 (100.0)	0.63
	At grandmother’s house	3 (6.8)	0	

* nonparametric distribution (verified using the Shapiro–Wilk test; *p* ≤ 0.05); ^1^ any missing data for respondents in the subgroup; ^2^ multiple-choice question; SD—standard deviation; n—number of children.
